# Lack of evidence for GDF11 as a rejuvenator of aged skeletal muscle satellite cells

**DOI:** 10.1111/acel.12475

**Published:** 2016-03-28

**Authors:** Aaron C. Hinken, Janine M. Powers, Guizhen Luo, Jason A. Holt, Andrew N. Billin, Alan J. Russell

**Affiliations:** ^1^ GlaxoSmithKlineMuscle Metabolism Discovery Performance Unit709 Swedeland RoadKing of PrussiaPA19406USA; ^2^ Five Prime TherapeuticsTwo Corporate DriveSouth San FranciscoCA94080USA

**Keywords:** Satellite cell, GDF11, aging

## Abstract

Recent high‐profile studies report GDF11 to be a key circulating ‘anti‐aging’ factor. However, a screen of extracellular proteins attempting to identify factors with ‘anti‐aging’ phenotypes in aged murine skeletal muscle satellite cells did not identify GDF11 activity. We have been unable to confirm the reported activity of GDF11, similar to other laboratories offering conflicting data and describe our attempts to do so in this short take.

Numerous reports have demonstrated the existence of factors present in the circulatory system of young rodents that can promote tissue regeneration in aged rodents (Lunsford *et al*., 1963; Conboy *et al*., 2005; Conboy *et al*., 2013). GDF11 was recently proposed to decline in concentration in old mice and to restore young tissue function phenotypes in the heart, CNS, and skeletal muscle (Loffredo *et al*., 2013; Sinha *et al*., 2014). It was proposed that GDF11 promotes skeletal muscle regeneration by restoring genomic integrity of old skeletal muscle satellite cells (SCs) and thereby promoting their outgrowth in response to injury (Sinha *et al*., 2014). To identify factors that may regulate the growth with aging, we screened a comprehensive expression library of extracellular proteins (Zhang *et al*., 2014) on aged skeletal muscle SCs (additional screen details to be published elsewhere). Given the reported activity of GDF11, we expected to identify it in our screen. However, this activity was not identified despite a GDF11 clone being present. To better understand the lack of GDF11 activity, we considered the conditions under which the screening assay was performed versus the conditions used by Sinha *et al*. Screening conditions used F10 basal medium supplemented with 20% heat‐inactivated horse serum (HI‐HS) and 25 ng/mL bFGF to support the outgrowth of SC cultures (Conboy *et al*., 2005). The studies published by Sinha *et al*. used a non‐mitogen‐containing medium for their experiments testing GDF11's ability to promote outgrowth of aged SCs (see Figure S19 in Sinha *et al*.). In addition, the authors used a modified culture system consisting of F10 plus 20% knockout serum replacement (KSR) and bFGF as the control condition and F10 plus 20% KSR and recombinant GDF11 (rGDF11) or recombinant GDF8 (rGDF8) with no bFGF as the test conditions. We therefore reasoned GDF11 was not identified in our screen because of these different culture conditions. We directly tested this hypothesis.

Commercially obtained rGDF11 was confirmed active and potent using a SMAD2/3 luciferase reporter assay (Fig. 1A), where rGDF11 exhibited an EC_50_ of ~67 pM (or ~4 ng/mL). Similarly, purchased rGDF8 was also active and potent in this assay. Modulation of SC outgrowth from young (3 months) or old mice (24 months) using conditions matching Sinha *et al*. was tested. rGDF8 reduced young SC numbers (Fig. 1B, *P *<* *0.05) consistent with published findings (Sinha *et al*., 2014; Egerman *et al*., 2015). However, we observed rGDF11 also tended to reduce young SC numbers (*P *< 0.05) in agreement with Egerman *et al*. but differing from the data reported by Sinha *et al*. that showed enhanced outgrowth. SC expansion was sustained by bFGF in young cultures (Fig. 1B, *P *< 0.001) as expected. Cultures of old SCs responded to bFGF, but none of the tested concentrations of rGDF8 or rGDF11 had an effect (Fig. 1B).

**Figure 1:**
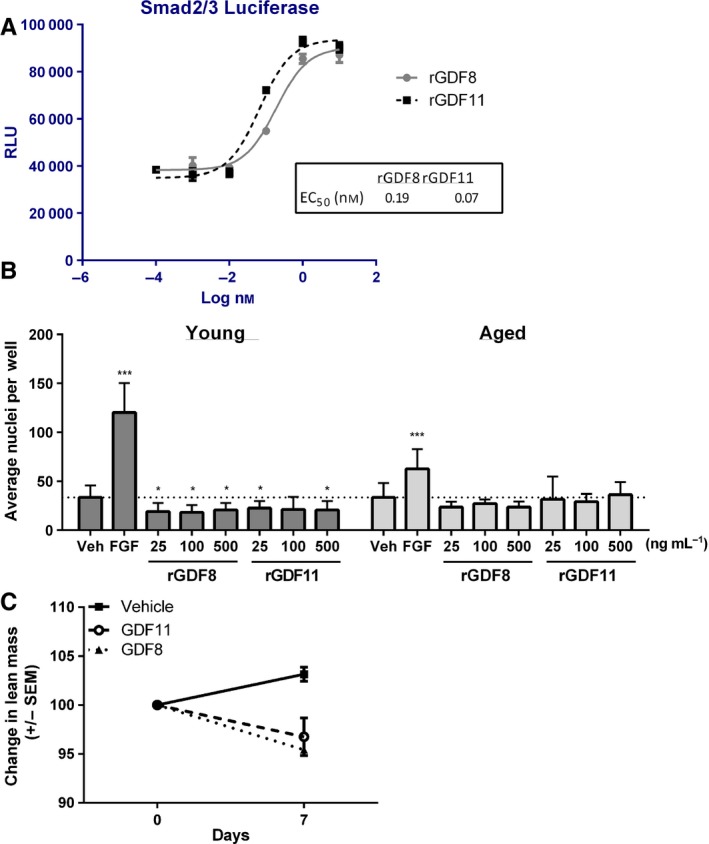
Similar activities of GDF8 and GDF11. (A) Purified rGDF11 and rGDF8 are potent and efficacious in a luciferase reporter gene assay of SMAD2/3 activity in HEK cells. (B) Satellite cells from young or aged animals were treated with rGDF11 or rGDF8 at various concentrations in F10 + 20% KSR. FGF stimulated outgrowth of both young and old cells. rGDF8 and rGDF11 had a modest negative effect on young satellite cell numbers and no effect in cultures of old satellite cells. Average values obtained from two distinct experiments (six replicates for each condition per experiment for a total of 12 wells) are shown (mean ± SD). *P* ‐values were calculated by Student's *t*‐test with statistically significant differences indicated (*P < 0.05, ***P < 0.001) matching methods by Sinha *et al*. (C) Expression of GDF8 or 11 in the liver by injection of expression vectors promotes rapid lean mass loss (*P *<* *0.05).

Interestingly, we observed SC outgrowth in the KSR‐containing media is not as robust as observed in HI‐HS (Bareja *et al*., 2014). This is different from the data by Sinha *et al*. in Figure S19 where SC outgrowth with bFGF was similar in KSR to that in HI‐HS media (*e.g*., 200 seeded cells growing out to 1000–2000 cells in approximately 5 days). We do not understand the basis for this difference between our observations versus those of Sinha *et al*.; however, our data suggest KSR media do not support survival and growth of the SCs as well as HI‐HS media, although the cells still respond to the mitogenic activity of bFGF.

Recent papers indicate GDF11 is not decreased in the circulation of aged rodents or older humans and that GDF11 is actually deleterious toward muscle repair in mice (Egerman *et al*., 2015; Rodgers & Eldridge, 2015). The experiments performed in Egerman *et al*. on isolated SCs demonstrated a small decrease in the number of cells after 3 days of rGDF8 or rGDF11 treatment. However, the conditions used for culture (DMEM with 20% horse serum) were different from those used by Sinha *et al*. as discussed above (see Table S1 for a comparison). The data we obtained using the culture system of Sinha *et al*. are most consistent with that presented by Egerman *et al*., at least with regard to the effects of rGDF11 on young SCs. In addition, we tested for the effects of both GDF8 and GDF11 on lean mass in young mice via expression in the liver by injection of expression plasmids containing GDF8 or GDF11 cDNA. Lean mass in these mice was reduced over a period of 7 days (Fig. 1C). These data are consistent with the fact that GDF11 shares a high degree of sequence identity with GDF8, activates similar signaling pathways (Lach‐Trifilieff *et al*., 2014), does not promote SC outgrowth, and reduces lean mass in mice. Thus in these findings, GDF11 exhibits activity similar to that of GDF8. Clearly, further investigation is needed to fully clarify whether, and under what, if any, conditions GDF11 is a factor that beneficially regulates muscle activity during aging.

## Supplemental materials

Animal Care and Models: For studies involving mice, all procedures performed were in compliance with the Animal Welfare Act and US Department of Agriculture regulations and approved by the GlaxoSmithKline or Five Prime Therapeutics Institutional Animal Care and Use Committee. For satellite cell isolations, mice were maintained on standard laboratory chow and allowed access to food and water *ad libitum*. Male C57Bl6/J were used for these experiments (Charles River Labs, Wilmington, MA, USA).

Satellite isolation and culture: Isolation and culture of murine SCs was performed as in (Bareja *et al*., 2014) and references therein. Cells were fixed, stained with Hoechst 33342 to visualize nuclei, and counted as previously described at the end of the culture period (Bareja *et al*., 2014). Recombinant mouse/rat/human GDF8 and GDF11 was obtained from R and D Systems and reconstituted according the manufacturer's directions.

### *In vivo* studies

Female BalbC mice (Charles River Labs) were randomly assigned into treatment groups (*n* = 10). On day 0, mice were subjected to lean mass analysis (EchoMRI^®^, Houston, TX, USA) to establish baseline measurements, then injected with expression vectors encoding GDF8, GDF11 or vehicle control and on day 7 lean mass was measured.

## Funding

No funding information provided.

## Conflict of interest

All authors are employees and/or stockholders of GlaxoSmithKline or Five Prime Therapeutics, Inc.

## Supporting information


**Table S1** Comparison of experimental conditions used in Sinha et al (2014) and Egerman et al. (2015).
