# Supplementary material for: Lack of evidence for GDF11 as a rejuvenator of aged skeletal muscle satellite cells
Source: Aging Cell. 2016 Mar 28;15(3):582–4. doi: 10.1111/acel.12475 (PMC4854912; doi:10.1111/acel.12475)
Supplement: Supplementary file 1 [file ACEL-15-582-s001.docx]

Supplemental Table 1 Comparison of experimental conditions used in Sinha et al (2014) and Egerman et al. (2015).

	Sinha et al.	Egerman et al.
Basal Medium	F10	DMEM
Serum	none	20% FBS
Serum Replacement	KSR	none
bFGF	Not present in GDF8 or GDF11 containing cultures	Yes, present in all culture conditions
Manuscript Figure number	S19	5
